# Pediatric Subspecialty Adoption of Telemedicine Amidst the COVID-19 Pandemic: An Early Descriptive Analysis

**DOI:** 10.3389/fped.2021.648631

**Published:** 2021-04-13

**Authors:** James Xie, Priya Prahalad, Tzielan C. Lee, Lindsay A. Stevens, Kara D. Meister

**Affiliations:** ^1^Division of Pediatric Anesthesiology, Department of Anesthesiology, Perioperative and Pain Medicine, Lucile Packard Children's Hospital—Stanford Children's Health, Stanford University School of Medicine, Stanford, CA, United States; ^2^Information Services Department, Stanford Children's Health, Stanford, CA, United States; ^3^Division of Pediatric Endocrinology and Diabetes, Department of Pediatrics, Lucile Packard Children's Hospital—Stanford Children's Health, Stanford University School of Medicine, Stanford, CA, United States; ^4^Division of Pediatric Rheumatology, Department of Pediatrics, Lucile Packard Children's Hospital—Stanford Children's Health, Stanford University School of Medicine, Stanford, CA, United States; ^5^Division of General Pediatrics, Department of Pediatrics, Lucile Packard Children's Hospital—Stanford Children's Health, Stanford University School of Medicine, Stanford, CA, United States; ^6^Division of Pediatric Otolaryngology, Department of Otolaryngology—Head and Neck Surgery, Lucile Packard Children's Hospital—Stanford Children's Health, Stanford University School of Medicine, Stanford, CA, United States

**Keywords:** telemedicine, telehealth, pediatric, subspecialty, access, COVID-19

## Abstract

Telemedicine has rapidly expanded in many aspects of pediatric care as a result of the COVID-19 pandemic. However, little is known about what factors may make pediatric subspeciality care more apt to long-term adoption of telemedicine. To better delineate the potential patient, provider, and subspecialty factors which may influence subspecialty adoption of telemedicine, we reviewed our institutional experience. The top 36 pediatric subspecialties at Stanford Children's Health were classified into high telemedicine adopters, low telemedicine adopters, and telemedicine reverters. Distance from the patient's home, primary language, insurance type, institutional factors such as wait times, and subspecialty-specific clinical differences correlated with differing patterns of telemedicine adoption. With greater awareness of these factors, institutions and providers can better guide patients in determining which care may be best suited for telemedicine and develop sustainable long-term telemedicine programming.

## Introduction

The COVID-19 pandemic has fueled the rapid implementation and adoption of telemedicine (TM) in pediatric care. In a pandemic, TM offers a unique venue to preserve patient access to care, while also providing a real-time benefit to public health via infection control by limiting patients' exposure to one another and providers (1, 2). Policymakers recognized the need and deregulated TM, accelerating its adoption and resulting in a national “telemedicine test case” (3). However, little is known about what kinds of pediatric patients are best served by digital modalities, which pediatric subspecialties are best suited to the adoption of TM, and what barriers might exist in the perpetuation of TM in pediatric subspecialty care. The rapid implementation of TM in response to the COVID-19 pandemic has provided a unique situation to study barriers, facilitators, and operational processes of TM in various pediatric subspecialties (4). As such, there have been a number of publications summarizing the experiences in various pediatric subspecialties such as endocrinology, medical genetics, and orthopedics (5–7). It is assumed that the effectiveness and durability of a TM program varies widely by pediatric specialty, patient population, and the preferences of patients and providers. To our knowledge, there have not been any publications summarizing a single institution's experiences across different pediatric subspecialties.

It is also known that differences in patient demographics such as race, language, insurance status, and neighborhood broadband status may impact the use of health-related technologies including patient portals and TM (8, 9). Inequities in accessing TM have also been reported in adult patients during the COVID-19 pandemic, with poorer, non-English-speaking, and Latinx patients having less TM use (10). To better delineate the potential patient, provider, and subspecialty factors influencing subspecialty adoption of TM, we studied a single institution's experience during the COVID-19 pandemic. We hypothesized that because subspecialties differ in the nature of the clinical encounter and needs, subspecialities within a single institution may have different TM adoption rates, and these differences may continue and evolve throughout the pandemic. Secondarily, we hypothesized that TM use may be driven by non-specialty factors including patient factors such as patient's preferred language (English speaking patients may be more likely to adopt to TM), insurance type (patients with non-public insurance may be more likely to adopt TM), and distance to the clinic (patients living farther away may be more likely to adopt TM), as well as institutional factors such as wait times to make appointments (subspecialties with long appointment wait times may be more likely to adopt TM).

## Materials and Methods

### Setting

Lucile Packard Children's Hospital/Stanford Children's Health is located in the San Francisco Bay Area, California. The quaternary academic teaching hospital, Lucile Packard Children's Hospital (LPCH), is located in Palo Alto, California, and is associated with Stanford School of Medicine. Stanford Children's Health (SCH) is comprised of more than 65 affiliated outpatient clinics and locations. Our institution was fortunate to have an existing TM platform, which although it had not previously been widely adopted, was quickly able to scale up and to have clinicians trained to use the platform in a short period of time. This led to a consistent adoption model across our institution.

Since our institution is in the San Francisco Bay Area, a local Shelter-in-Place (SIP) order went into effect on March 16, 2020, followed by a California SIP on March 19, 2020. An institution-wide request to convert appropriate in-person encounters to TM encounters was issued starting March 15, 2020. The hospital and providers opted to limit in-person visits when not necessary, and many patients and families did not want to be exposed unnecessarily. For the majority of the study period, Santa Clara County, the county in which Stanford is situated, remained at the highest risk tier of purple with only a brief decrease to the second highest tier in September, 2020. No specific restrictions were imposed on patients and families desiring to seek medical care during the study period.

### Data Acquisition

Data for outpatient clinical encounters was queried from the electronic medical record, ambulatory access dashboards, and billing databases at LPCH/SCH. TM encounter data from ambulatory clinic visits from January 1, 2020, through November 15, 2020, was obtained with associated data on each patient's primary language, home zip code, insurance type, clinic specialty, and provider identification number. Of note, a pediatric patient's language is recorded with the parent or caregiver's preferred language. A linear distance from the center of the patient's home zip code to the Stanford, CA zip code (94305) was calculated to estimate proximity to SCH, where the majority of clinics are located. Wait times for clinic appointments were averaged over the study period to account for changes in wait times during the pandemic.

### Statistical Analysis

To compare TM adoption patterns across subspecialties, three groups were defined by using a “simple majority” cutoff of 50% of visits being telehealth: (1) Low TM adopters (clinics that never increased share of TM > 50% vs. in-person visits in 2020); (2) High TM adopters (clinics that increased share of TM > 50% vs. in-person and remained > 50% for the rest of the year); and (3) TM reverters (clinics that increased TM > 50% vs. in-person but fell back to < 50% TM shortly thereafter).

Demographic and clinical characteristics were statistically analyzed. Analysis was performed on a visit-level basis (i.e., each TM visit was weighted the same even if the same patient had multiple visits). Descriptive statistics were used to examine and highlight trends. Numerical data was expressed as mean and standard deviation, and categorical variables were expressed as absolute frequencies and percentages. The proportion of patients with public vs. managed care insurance was compared for the top four language groups of our patients. In order to compare TM adoption groups, we applied paired *t*-test for numerical parameters and Chi Square test with Yates' continuity correction for categorical variables. One-way ANOVA with Tukey's Honestly Significant Difference (HSD) *post-hoc* test was used when the means of more than two groups were being analyzed. A *p* < 0.05 was considered statistically significant.

## Results

### Subspecialties With TM Visits

**Figure 1:**
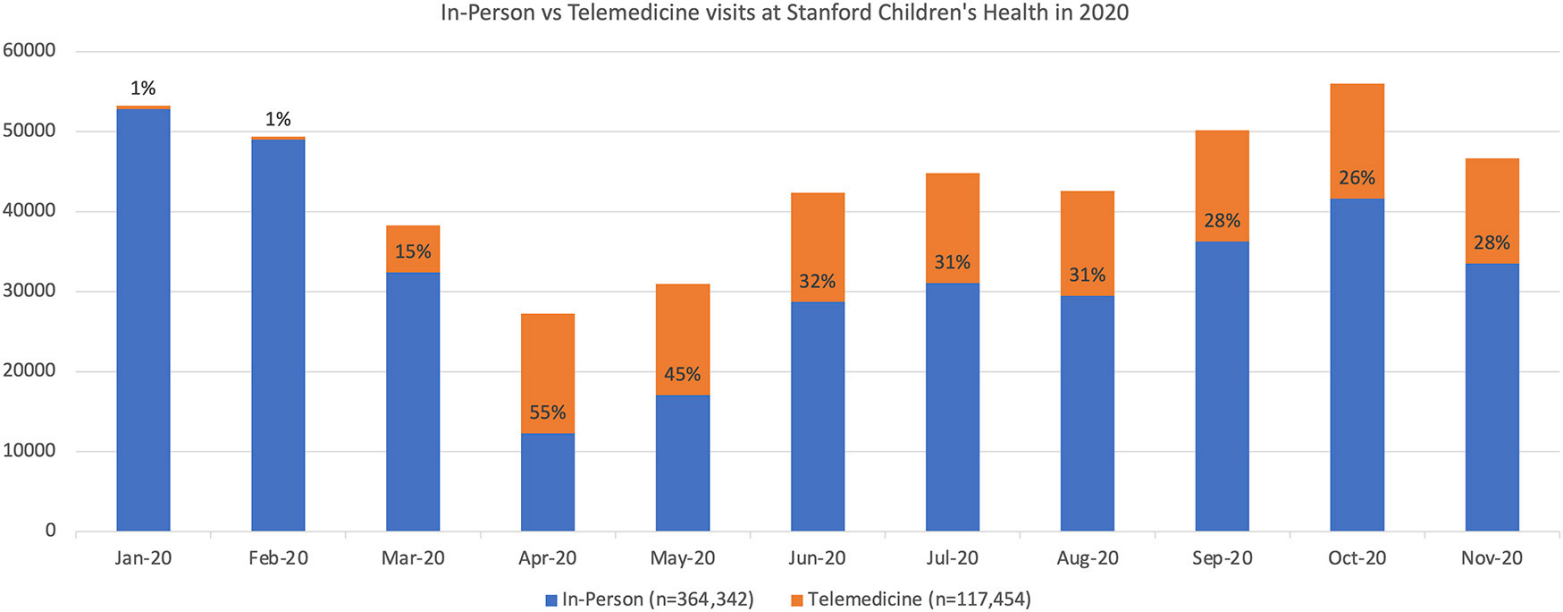
Percentage of In-person vs. TM visits at Stanford Children's Health in 2020.

Across our institution, TM visits increased in quantity for every subspecialty during 2020. A summary of in-person vs. TM visits for 36 subspecialties and general pediatrics at SCH in 2020 is depicted in Figure 1. Prior to March 2020, there were very few TM visits at our institution (i.e., <1% of all clinic encounters, from January 2016 to February 2020 there were 6,305 TM visits from 2,344 unique patients). From March 2020 to November 2020, there were 123,416 TM visits from 72,819 unique patients. April 2020 saw the highest total number of TM visits at 14,938; April 2020 was also unique in that TM visits exceeded in-person visits (*n* = 12,302) across our institution. Peak TM monthly percentages ranged from 18.2% (Cardiology, April 2020) to 100% (Weight Management, April 2020).

There were 28 subspecialties with >50% TM visits in March/April/May 2020. Of these, 12 subspecialties maintained >50% TM visits for the remainder of 2020. These subspecialties were considered “high TM adopters.” There were nine subspecialties with <50% TM visits in March/April/May 2020 and beyond; these were considered “low TM adopters.” There were 16 subspecialties that returned to <50% TM visits after May 2020; these subspecialties were considered “TM reverters.” These subspecialties are listed in Table 1.

**Table 1:** Pediatric subspecialty telemedicine use by adoption pattern.

**Subspecialty**	**Peak TM %**	**Peak TM month**	
**Low TM adopters: Subspecialties with** ** <50% TM visits March/April/May 2020 and beyond**			
Adolescent medicine	20.7%	May-20	
Cardiology	18.2%	April-20	
Hand surgery	28.6%	May-20	
Hematology	27.4%	April-20	
Oncology	25.1%	April-20	
Ophthalmology	28.0%	April-20	
General pediatrics	46.3%	April-20	
Plastic surgery	46.8%	April-20	
Stem cell transplant	26.7%	April-20	
**Subspecialty**	**Peak TM %**	**Peak TM month**	
**High TM adopters: Subspecialties which maintained** **>50% TM visits for rest of 2020**			
Developmental behavioral pediatrics	98.3%	May-20	
Diabetes	95.1%	April-20	
Eating disorders	97.7%	April-20	
Gastroenterology	80.7%	April-20	
Gender	98.4%	April-20	
Genetics	84.6%	April-20	
Immune behavioral health	95.4%	April-20	
Liver transplant	73.6%	May-20	
Neurology	96.4%	April-20	
Pain medicine	98.6%	April-20	
Psychiatry	99.0%	July-20	
**Subspecialty**	**Peak TM %**	**Peak TM month**	**Month when TM returned to** ** <50%**
**TM reverters: Subspecialties that returned to** ** <50% TM visits beyond May 2020**			
Allergy and immunology	57.0%	April-20	May-20
Cardiovascular transplant	71.2%	April-20	May-20
Cystic fibrosis	75.7%	April-20	May-20
Dermatology	98.2%	April-20	August-20
Endocrinology	91.3%	April-20	November-20
General surgery	75.0%	April-20	May-20
Gynecology	71.6%	April-20	June-20
Infectious diseases	72.2%	April-20	June-20
Nephrology	96.6%	April-20	June-20
Neuro-oncology	57.1%	April-20	May-20
Neurosurgery	68.6%	April-20	September-20
Orthopedics and sports medicine	63.2%	April-20	May-20
Otolaryngology	71.5%	April-20	May-20
Pulmonology	94.7%	April-20	June-20
Rheumatology	94.8%	April-20	July-20
Urology	57.8%	April-20	May-20

*There were 36 subspecialities examined across our institution. The subspecialties were divided into three cohorts based on rate of TM adoption. Subspecialties which maintained >50% TM visits for the remainder of 2020 were considered “high TM adopters.” Subspecialties which did not reach >50% TM visits during any month in 2020 were considered “low TM adopters.” There were 16 subspecialties that initially had >50% TM visits in early 2020, but then returned to <50% TM visits beyond May 2020; these subspecialties were considered “TM reverters”*.

### Patient Insurance Among Subspecialty TM Visits

The proportion of visits with public insurance for low TM adopters, high TM adopters, and TM reverters is shown in Table 2. Low TM adopters had a lower percentage (21.7%) of public insurance compared to high TM adopters (25.7%), χ^2^ = 165.3, *p* < 0.01, Odds ratio (OR) of 1.25 (95% CI: 1.21–1.30) of having managed care if a patient was in the low TM adopter group relative to high TM group, and a lower percentage of public insurance compared to TM reverters (33.3%), χ^2^ = 900.4, *p* < 0.01, OR = 1.80 (95% CI: 1.73–1.87). The difference between high TM adopters and TM reverters was also significant χ^2^ = 468.9, *p* < 0.01, OR = 1.44 (95% CI: 1.39–1.49).

**Table 2:** Patient factors among pediatric subspecialty TM visits.

	**Low TM adopters**	**High TM adopters**	**TM reverters**
	***n* (%)**	***n* (%)**	***n* (%)**
Managed Care	22,467 (78.3)	38,845 (74.3)	16,344 (66.7)
Public Insurance	6,222 (21.7), **(a)** ***p*** **<** **0.01**	13,466 (25.7)	8,154 (33.3), **(a)** ***p*** **<** **0.01**
English Language	27,563 (95.1), **(b)** ***p*** **<** **0.01**	48,145 (90.4)	21,776 (87.7), **(b)** ***p*** **<** **0.01**
Non-English Language	1,424 (4.9)	5,113 (9.6)	3,062 (12.3)
	**Mean (miles)**	**Mean (miles)**	**Mean (miles)**
Distance to home	39.07, **(c)** ***p*** **<** **0.001**	53.09, **(c)** ***p*** **<** **0.001**	64.92, **(c)** ***p*** **<** **0.001**

*(a) Low TM adopters were more likely to have managed care insurance than high TM adopters [OR = 1.25 (95% CI: 1.21–1.30), p < 0.01], or TM reverters [OR = 1.80 (95% CI: 1.73–1.87], p < 0.01]. (b) Low TM adopters were more likely to be English speaking than high TM adopters [OR = 2.06 (95% CI: 1.93–2.19), p < 0.01] or TM reverters [OR = 2.72 (95% CI: 2.55–2.91), p < 0.01]. (c) There was a significant difference among the three groups (low TM adoption, sustained high TM adoption, and TM adopters that reverted back to >50% in-person visits) for linear distance between the patient's zip code and Stanford, CA, p < 0.001, with low TM adopters having the shortest mean distance*.

### Primary Language Among Subspecialty TM Visits

There were statistically different rates of English-preferred language patients vs. non-English-preferred language patients among the three groups of subspecialty TM adoption. The largest differences were seen between the low TM adopters and high TM adopters and the low TM adopters and TM reverters. Low TM adoption was associated with a higher percentage (95.1%) of English-preferred language patients when compared to high TM adopters (90.4%),χ^2^(1, *N* = 53,258) = 563.17, *p* < 0.01, OR = 2.06 (95% CI: 1.93–2.19), and TM reverters (87.7%), χ^2^(1, *N* = 24,838) = 961.79, *p* < 0.01, OR = 2.72 (95% CI: 2.55–2.91).

### Primary Language by Insurance Type

Across TM use at our institution, insurance type differs by preferred language as summarized in Table 3. 97.5% of managed care patients were English-preferred language patients while 70.8% of public insurance patients were English-preferred language patients. Spanish-preferred language patients had a significantly higher percentage of patients with public health insurance (90.8% with public insurance) compared to English-preferred language patients (20.2% with public insurance), χ^2^[(1, *N* = 92,280) = 22,200, *p* < 0.001].

**Table 3:** Preferred language and insurance type.

**Patient's preferred language**	**Managed care**	**Public insurance**		
	***n* (%)**	***n* (%)**		
English	91,433 (80%)	23,245 (20%)		
Spanish	847 (9%) **(a)** ***p*** **<** **0.001**	8,318 (91%)		
Chinese (Mandarin or Cantonese)	654 (57%) **(b)** ***p*** **<** **0.001**	498 (43%)		
Other language	800 (51%) **(c)** ***p*** **<** **0.001**	771 (49%)		
**Insurance type**	**English**	**Spanish**	**Chinese (Mandarin or Cantonese)**	**Other language**
	***n*** **(%)**	***n*** **(%)**	***n*** **(%)**	***n*** **(%)**
Managed care	91,433 (97.5%)	847 (0.9%)	654 (0.7%)	800 (0.9%)
Public insurance	23,245 (70.8%)	8,318 (25.3%)	498 (1.5%)	771 (2.4%)

*Patient's preferred language relative to patient's insurance type for all telemedicine encounters. (a) Primarily English-speaking patients have a significantly higher percentage of patients with managed care insurance than Spanish-speaking patients [χ^2^(1, N = 92,280) = 22,200, p < 0.001], (b) Mandarin/Cantonese-speaking patients [χ^2^(1, N = 92,087) = 367.5, p < 0.001], or (c) those patients speaking other languages [χ^2^(1, N = 92,233) = 782.9, p < 0.001]*.

### Distance to Home Comparison Among Subspecialty TM Visits

The linear distance from patient's home zip code to Stanford, CA was used as an approximation of travel burden. The average distances had a right-skewed distribution: low TM adopters had a mean distance of 39.1 miles, median 24.2 miles, SD 127.5 miles; high TM adopters had a mean distance of 53.1 miles, median 21.0 miles, SD 157.5 miles; TM reverters had a mean distance of 64.9 miles, median 27.1 miles, *SD* = 213.5 miles. A one-way ANOVA showed the differences in mean distance traveled from home to clinic was significant [*F*_(2)_ = 165.74, *p* < 0.001]. *Post-hoc* analyses using the Tukey HSD *post-hoc* test indicated that the mean distance traveled from home to clinic was significantly lower in the low TM adopters (*M* = 39.07) than in the high TM adopters (*M* = 53.09) and the TM reverters (*M* = 64.92).

### Clinic Wait Time Among Subspecialties

When compared to low TM adopters (*M* = 5.31 days, *SD* = 4.91), high TM adopters (*M* = 18.33 days, *SD* = 15.06) had a longer average number of days from referral to first visit scheduled [*t*_(15)_ = 2.33, *p* = 0.03].

## Discussion

The COVID-19 pandemic forced the use of TM in many pediatric subspecialties during the early months of the pandemic, including some subspecialties for which TM was previously seen as unviable. The proportion of TM visits in clinics with pre-pandemic adoption of TM also rose rapidly. However, some subspecialties were low utilizers of TM, and others shifted back to majority in-person visits relatively quickly. Understanding the patient factors, provider/institutional factors, and subspecialty clinical factors which may drive TM use will enable institutions to develop more effective digital health programs. Our data demonstrates there are multiple factors which correlate with whether a subspecialty adopts and sustains high rates of TM encounters.

Overall, there was an initial reduction in total visit numbers at our institution, as reflected in Figure 1. The decrease in visits, especially in March/April 2020, is likely multifactorial and likely includes a combination of: (1) families not wanting to be exposed to COVID-19, (2) less exposure to common infections requiring care, (3) SIP/social distancing protocols, and (4) downstream effects of decreased referrals from community providers as fewer patients were being seen by general practitioners.

Patient factors, including insurance type, preferred language, and distance from home to clinic, were different between low TM adopter subspecialties and other subspecialties. For insurance type, although there are statistically significant differences among the three TM adoption pattern groups, none of the odds ratios are >2, suggesting a relatively weak association. Of note, California has had payor parity for telemedicine since 2019 following the signing of AB744 which mandates that payors reimburse healthcare providers for telehealth services “on the same basis and to the same extent” as they cover in-person services (11). This may explain why the proportion of patients with public insurance remained approximately consistent with our institution's internal data on payor mix prior to the COVID-19 pandemic.

While it is not possible to directly attribute TM adoption rates to patients' preferred language, our findings showed a lower percentage of non-English-preferred language patients in clinics with low TM adoption. Only 4.9% of TM visits in low adoption specialties were with non-English-preferred language patients. This is lower than expected compared to the pre-pandemic language mix in those clinics. This may indicate that when given the option for in-person visits or TM, non-English-preferred language patients chose in-person. This finding may also be related to the need for TM-enabled interpreter services and the need to set up a third-party interpreter during a TM visit. In-person visits have a more established interpreter workflow and thus perhaps fewer non-English-preferred language patients were being seen via TM in the low TM adoption group. Equitable accessibility for all patients is challenging (12, 13). Families need solutions in their preferred language, at their level of health literacy, and digital literacy. Patients experiencing healthcare disparities show less engagement in telehealth, including use of patient portals and TM visits (14, 15). Despite being situated in the Silicon Valley, our institution still sees some of the most explicit examples of the “digital divide” in our families (16).

Similarly, low TM adoption was associated with a shorter mean distance from home to the clinic. This finding could reflect that subspecialties with a regional catchment (e.g., cardiology, hematology, and oncology) remained low TM adopters due to the inherent proximity of their patients. Clinics that maintained high TM rates were able to see more patients farther away. For the clinics that reverted back to >50% in-person visits, the visits that remained TM may have been for patients who live farther away. This may suggest that patients were more willing to travel to an in-person encounter when the distance, and corresponding burdens of travel and time, was less. Conversely, in the high TM adopters and remaining TM visits in the reverter group, the potential disadvantages of TM (unfamiliarity, technology, and accessibility needs) may have been outweighed by the convenience and option to not travel. Patients in rural counties have been shown to be more likely than their urban counterparts to use TM for pediatric rheumatology care (17), high risk obstetrics (18), and otolaryngology (19); similarly, TM for pediatric neurosurgery care has been shown to be feasible and save families substantial travel time, travel cost, and time away from work (20). Among pediatric Medicaid beneficiaries, TM use is more likely in rural children (12). In addition, rural pediatricians have also expressed enthusiasm about telehealth strategies to improve access to subspecialty care (21).

Provider/institutional factors, such as wait time, also may influence long-term TM adoption (22). As we embarked on evaluating the changes in TM adoption in our subspecialty clinics, we hypothesized that clinics with longer wait times may be more likely to continue with TM to help improve access to care. In our data, subspecialties that were high TM adopters had longer times between referral to first visit than low TM adopters. This suggests that subspecialties with a longer average wait time to be seen were more inclined to transition to TM and sustain TM appointments beyond May 2020. As TM can maximize usage of physical space and provider productivity, it is logical that institutions may prioritize digital health as an avenue to increase access, especially in those subspecialties with more constrained schedules. Alternatively, the finding that high TM adopters had longer times between referral to first visit may indicate that patients were willing to transition to TM rather than cancel or reschedule a long-awaited appointment in these subspecialties.

Provider willingness to adopt and sustain TM, providers' comfort with resuming in-person visits, adaptability of scheduling algorithms, technical readiness, and other factors not captured in the presented data should also be considered (23). Our institution was fortunate to deploy a multi-lingual TM platform within our EHR-embedded patient portal across subspecialties and had institutional on-demand technical support and training for TM encounters. The method by which appointments were converted from in-person to TM was variable by pediatric specialty. Scheduling algorithms and call centers differ between subspecialties at our institution, making access a significant potential confounder to TM adoption between subspecialties.

There are likely inherent differences in the clinical encounters of different subspecialties which strongly influenced TM adoption. These include visits with a linked or connected service or study, specific physical examination techniques or perceived reliance upon the physical examination (e.g., slit lamp exam for ophthalmology, joint exam for rheumatology), proportion of patients with high-risk diagnoses (oncology, stem cell transplant), and the appropriateness and ratio of new vs. established encounters. At our institution, high TM adopters were non-surgical subspecialties with the exception of liver transplant (which includes a large number of pre-transplant and post-transplant medical visits and has a long-standing TM program for patients, local physicians, and transplant coordinators). In our early experience, those patients needing a linked or connected service, such as the cardiology patient needing an echocardiogram or the hand surgery patient needing an x-ray, had lower utilization of TM appointments. In addition, those specialties that rely heavily on the physical examination were slower to adopt the TM platform. Adoption in rheumatology and otolaryngology TM visits were seemingly born out of necessity—both specialties had very few or no TM encounters prior to the COVID-19 pandemic, because the physical exam is crucial to decision making. Within otolaryngology, there may be subspecialties that are more amenable to TM, such as the evaluation of tonsillar hypertrophy which can be visualized with basic video tools, rather than middle ear pathology, which requires specialized equipment for examination.

There are several limitations to the presented data, most notably in aspects of healthcare delivery which are not included in our dataset. Analysis of the in-person visits before and during the study period, as well as the visit types (new vs. established) would be helpful to provide granularity and examine the influence of encounter types on TM adoption. There may be a baseline difference between subspecialties in the proportion of new vs. established visits offered via TM. Unfortunately, subspecialty clinics have significant variability in differentiating between new and follow-up encounters, so we were unable to account for these differences. Moreover, by using visit-level data in our analysis, there will be a natural representation bias, skewing the demographic data toward patients who had multiple visits via TM (although as we show in our results, the 123,416 TM visits from March 2020 to November 2020 represented 72,819 unique patients). Our categorization schema for low vs. high TM adoption relies on a simple majority, as definitions of operationally or clinically significant rates of TM adoption are currently lacking. In the analysis of distance to clinic, a linear distance was used between the patient's zip code and Stanford, CA. This approach should be treated as a rough approximation as it does not reflect estimated driving time and is calculated by zip codes which cover larger geographies in rural areas. Importantly, we do not discuss any patient preference or patient experience data, and how that may have influenced TM rates over the course of the year. Early data from our institution suggests there are novel concerns in patient acceptability of pediatric TM experiences, such as the role for the caregiver (24). Similarly, provider experience data and no-show/late cancellation data by subspecialty would be necessary in developing a robust TM program. With regards to clinical appropriateness, we do not have any data on conversions to in-person visit or admission within an interval of the TM visit, which could be indicative of duplicative care or an inappropriate initial triage to TM. In some instances, TM was used in triage to determine if an in-person visit was necessary despite shelter-in-place guidance. In short, the data presented may be beneficial in delineating which subspecialties are best suited to developing sustained TM programs and exploring factors driving TM persistence, but it cannot robustly determine whether the clinical goals of patient care and patient/provider experiences are being met.

There has undoubtedly been progress in TM and digital health in pediatric subspecialty care driven by adaptation to the constraints of the COVID-19 pandemic. To solidify this progress, institutions must further define goals for TM adoption for each subspecialty to address. Some aspects may be consistent across subspecialties, such as ensuring equity in access for patients of all languages and socioeconomic backgrounds or defining criteria for essential in-person visits. Other aspects of TM adoption may be subspecialty specific, such as how to handle reliance upon physical examination or the need for a connected services (25). Subspecialty programs with low TM adoption may look toward innovations to help overcome barriers, such as a digital stethoscope in cardiology, but this should be done in the context of overall appropriateness of TM to the subspecialty and the patients it serves. High TM adopters may also benefit from re-evaluating the patient populations, diagnoses, and experiences of their patients to better design workflows and fine-tune clinical encounters for TM. Overall, clinical appropriateness criteria for TM and in-person visits will need to be validated prospectively. This could help guide an institutions' approach to ambulatory care models for pediatric subspecialty care models. By looking at these characteristics, a model could be created to predict volume and using some of these factors that may drive resource allocation for program development. By continually analyzing patient-based and systems-based data, we can optimize the positive impact of TM across pediatric subspecialty care.

## Data Availability Statement

The raw data supporting the conclusions of this article will be made available by the authors, without undue reservation.

## Author Contributions

JX: concept, data retrieval, data analysis, data interpretation, review and editing of manuscript, and final approval of manuscript. PP: concept, data interpretation, and final approval of manuscript. TL: concept, data retrieval, data interpretation, and review and editing of manuscript, and final approval of manuscript. LS: concept, data interpretation, and final approval of manuscript. KM: concept, data retrieval, data analysis, data interpretation, drafting of initial manuscript, review and editing of manuscript, and final approval of manuscript. All authors contributed to the article and approved the submitted version.

## Conflict of Interest

The authors declare that the research was conducted in the absence of any commercial or financial relationships that could be construed as a potential conflict of interest.
